# Zn/Cr-MOFs/TiO_2_ Composites as Adsorbents for Levofloxacin Hydrochloride Removal

**DOI:** 10.3390/molecules29184477

**Published:** 2024-09-20

**Authors:** Fuhua Wei, Qin Zhang, Qinhui Ren, Hongliang Chen, Yutao Zhang, Zhao Liang

**Affiliations:** 1 College of Chemistry and Chemical Engineering, Anshun University, Anshun 561000, China; 18208464727@163.com (Q.Z.); rqh285554560@163.com (Q.R.); zyt0516@126.com (Y.Z.); 2 Institute of Micro/Nano Materials and Devices, Ningbo University of Technology, Ningbo 315211, China

**Keywords:** MOFs, composites, adsorption, antibiotic

## Abstract

The Zn/Cr-MOFs/TiO_2_ composites were synthesized using the solvothermal method. XRD, FTIR, and SEM techniques were utilized to characterize the Zn/Cr-MOFs/TiO_2_ composites employed for simulating levofloxacin hydrochloride in wastewater. The impact of the mass of the Zn/Cr-MOFs/TiO_2_ composite, concentration of levofloxacin hydrochloride, solution pH, and temperature on the adsorption performance was investigated. Experimental findings indicated that at pH 6, the maximum removal efficiency of levofloxacin hydrochloride by the Zn/Cr-MOFs/TiO_2_ composite was achieved at 88.8%, with an adsorption capacity of 246.3 mg/g. To analyze the experimental data, both pseudo-first-order and pseudo-second-order kinetics models were applied, revealing that the pseudo-second-order model provided a better fit to the data. Additionally, Langmuir and Freundlich isotherm models were used to study equilibrium adsorption behavior and showed good agreement with both kinetic modeling and Langmuir isotherm analysis results. These observations suggest that monolayer adsorption predominates during the removal process of levofloxacin hydrochloride by Zn/Cr-MOFs/TiO_2_ composites.

## 1. Introduction

In recent years, the rapid progress of urbanization and industrialization has led to a range of environmental and energy challenges, including climate change, air and water pollution, and resource depletion [1,2]. The degradation of environmental quality emphasizes the need to bridge the gap between development and sustainability, with a significant amount of pollutants entering aquatic ecosystems [3]. Among the various types of heterogeneous organisms introduced into the environment, antibiotics are commonly detected as organic contaminants in wastewater. To address these challenges effectively, different innovative materials have been utilized to mitigate their impact [4,5,6], with particular emphasis on adsorption [7,8,9] and photocatalysis [10] due to their environmentally friendly nature. Adsorption is known for its simplicity, efficiency, and cost-effectiveness. Conversely, when solid materials act as photocatalysts, utilizing solar energy to decompose organic pollutants, photocatalysis proves highly advantageous. Metal–organic frameworks (MOFs) are recognized as an optimal selection for achieving this objective, attributable to their distinctive characteristics such as a substantial specific surface area and a porous architecture [11].

MOFs have demonstrated effectiveness in solid absorption media applications, including dye removal, due to their high porosity and adjustable chemical composition and structure [12,13,14,15]. However, MOFs’ negative charge under neutral conditions limits their potential as adsorbents for anionic chemicals, necessitating chemical modification [16,17,18]. TiO_2_ is a widely utilized photocatalyst with desirable properties such as high photocatalytic activity and non-toxicity [19,20]. Among various polycrystalline forms of TiO_2_, anatase is preferred primarily because of its superior charge handling performance [21]. Although pure TiO_2_ exhibits lower adsorption capacity compared to MOF structures, modifying it with MOFs creates a core–shell structure that enhances the ability to extract photogenerated electrons and holes from titanium dioxide by creating a heterojunction between MOFs and titanium dioxide [22,23]. The integration of Metal–Organic Frameworks (MOFs) with TiO_2_ has garnered significant interest in the realm of photocatalysis. Specifically, TiO_2_@UiO-66 has been employed for the catalytic conversion of CO_2_ into CH_4_ [24], dimethyl sulfide [25], organic dyes [26,27], and the mineralization of volatile organic compounds [28]. Additionally, TiO_2_@UiO-66-NH2 has shown utility in the reduction of Cr(VI) and the degradation of bisphenol A [29], as well as the breakdown of toluene and ether [30]. The r PMOF-55@TiO_2_ and PMOF-56@TiO_2_ nanocomposites have been developed for the degradation of non-steroidal anti-inflammatory drugs (NSAIDs) [31], while 2D M-TCPP MOFs/TiO_2_ have been investigated for the mineralization of organic dyes [32].

Considering the aforementioned factors, Zn/Cr-MOFs/TiO_2_ composites were synthesized by employing zinc acetate and chromium acetate as metal ion sources, along with 2,5-dihydroxyterephthalic acid as the organic coordination system and TiO_2_ by the one-pot method. Our findings indicate that the superior adsorptive performance is attributable to the enhanced charge separation facilitated by the synergistic interaction of metal ions, along with the cumulative effect of the enriched electron domain heterojunctions within the adsorbent matrix, which collectively enable effective removal of antibiotics. Furthermore, we have employed Zn/Cr-MOFs/TiO_2_ composites as photosensitive adsorbents to investigate the influence of antibiotic concentration, pH conditions, and adsorbent mass on the adsorption kinetics. A proposed mechanism elucidates the visible-light-driven adsorption of antibiotics onto Zn/Cr-MOFs/TiO_2_ composites.

## 2. Results and Discussion

### 2.1. Material Characterization

**Figure 1:**
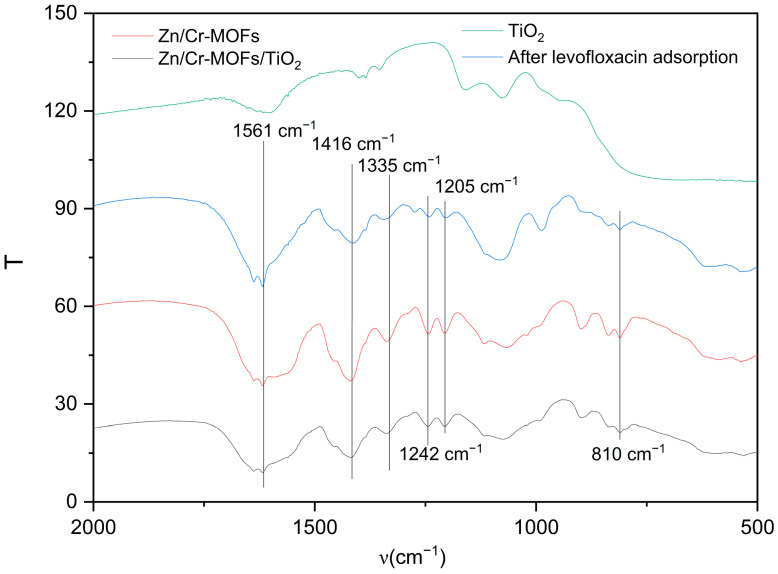
FTIR of Zn/Cr-MOFs/TiO_2_.

**Figure 2:**
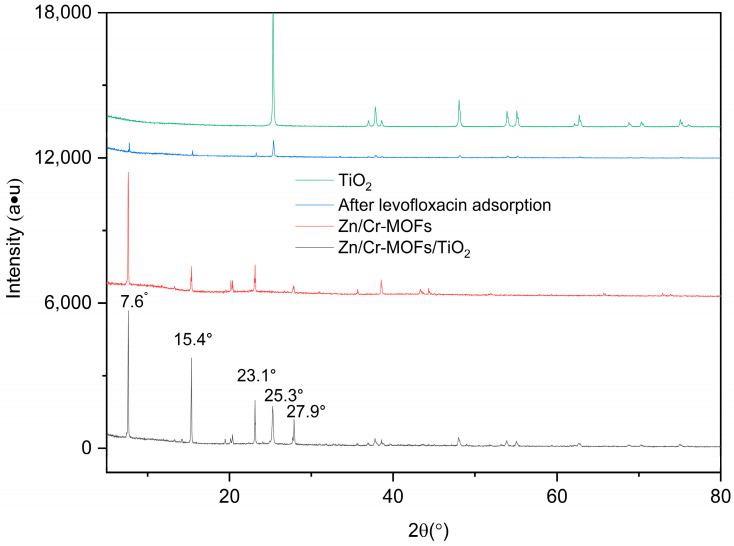
XRD of Zn/Cr-MOFs/TiO_2_.

**Figure 3:**
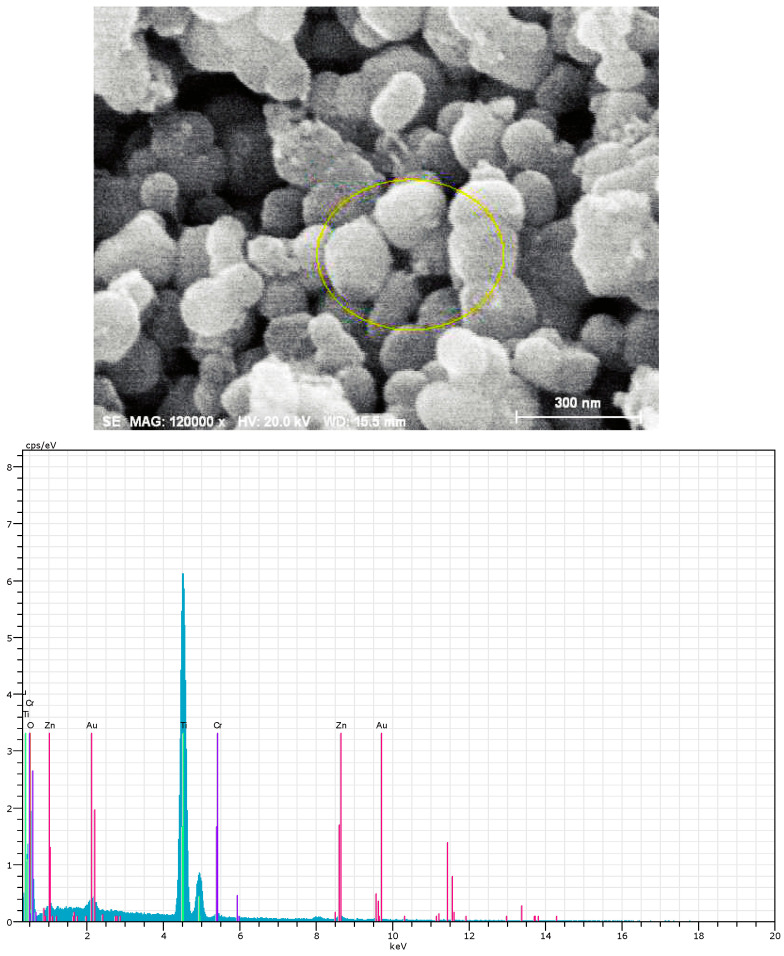
SEM and Atomic energy spectra of Zn/Cr-MOFs/TiO_2_.

**Figure 4:**
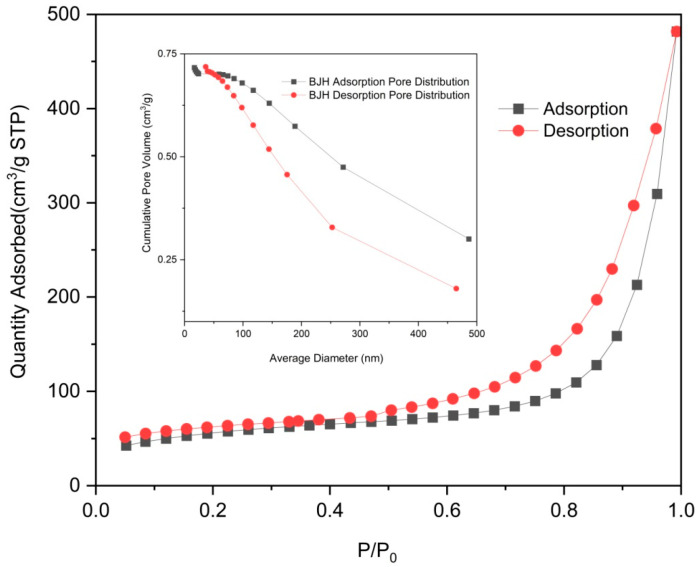
N_2_ adsorption–desorption isotherms of Zn/Cr-MOFs/TiO_2_.

The absorption peaks at 1416 cm^−1^ and 1561 cm^−1^ observed in Zn/Cr-MOFs/TiO_2_ (Figure 1) can be attributed to the delocalization of carboxyl groups in the organic ligand, resulting in the formation of two equivalent C-O bonds [33]. In the infrared spectrum, the peak at 1335 cm^−1^ is attributed to the carbonyl stretching vibration of the C-O bond. The absorption features at 1242 cm^−1^ and 1205 cm^−1^ are assigned to the out-of-plane rocking mode of the CH_2_ group, while the peak at 810 cm^−1^ corresponds to the substitutional absorption of the benzene ring. Figure 2 illustrates that Zn/Cr-MOFs/TiO_2_ exhibits distinct absorption bands at 7.6°, 15.4°, 23.1°, 25.3°, and 27.9°, with 25.3° being the predominant absorption peak of TiO_2_. Upon adsorption of levofloxacin, a reduction in the intensity of these absorption features was observed. As depicted in Figure 3 on account of the inherent poor electrical conductivity of Zn/Cr-MOFs/TiO_2_, the presence of gold nanoparticles is evidenced in the energy-dispersive X-ray (Figure 2) spectrum subsequent to gold sputtering. The high crystallinity of Zn/Cr-MOFs/TiO_2_, as evidenced by Figure 2 and Figure 3, is a consequence of enhanced molecular interaction among organic ligands that effectively promotes crystal growth in solvents. Furthermore, Zn/Cr-MOFs/TiO_2_ exhibits favorable morphology, structure, and dispersion characteristics. Analysis of N_2_ adsorption–desorption isotherms (Figure 4) reveals a Brunauer–Emmet–Teller-specific surface area of 192 m^2^/g for Zn/Cr-MOFs/TiO_2_ with an average particle size measuring approximately 17 nm. These findings indicate the mesoporous properties possessed by this material.

### 2.2. Removal of Levofloxacin Hydrochloride by Zn/Cr-MOFs/TiO_2_ Composites

**Figure 5:**
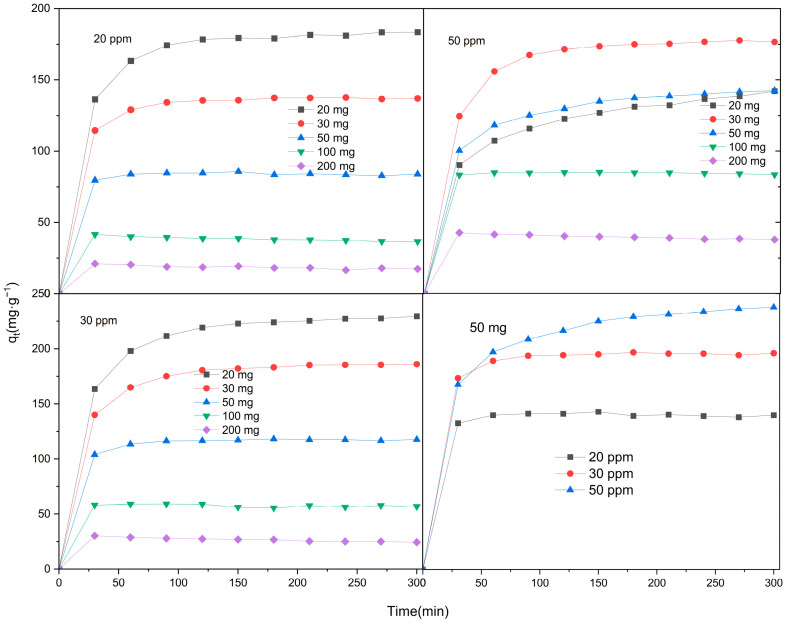
Adsorption capacity of levofloxacin by Zn/Cr-MOFs/TiO_2_. (The errors of concentration and mass are less than ±5% and ±1%, respectively.)

To investigate the adsorption characteristics of Zn/Cr-MOFs/TiO_2_ on levofloxacin hydrochloride, a comprehensive investigation was conducted by manipulating the dosage of Zn/Cr-MOFs/TiO_2_, the concentration of levofloxacin hydrochloride solution, and the adsorption time. As illustrated in Figure 5, when utilizing 200 mg of Zn/Cr-MOFs/TiO_2_ with a concentration level set at 20 ppm for levofloxacin hydrochloride solution, maximum adsorption capacity reached its peak value at 21 mg/g within just 30 min due to excessive coverage on active sites caused by an excessive quantity of Zn/Cr-MOFs/TiO_2_. However, when employing 50 mg of Zn/Cr-MOFs/TiO_2_ and setting the concentration of levofloxacin hydrochloride solution at 20 ppm, an impressive removal rate of 88.8% for levofloxacin hydrochloride was achieved within a duration of 150 min. Furthermore, experimental findings demonstrated that increasing the amount of Zn/Cr-MOFs/TiO_2_ while maintaining a constant concentration level for levofloxacin hydrochloride resulted in higher removal rates. Conversely, keeping the dosage of Zn/Cr-MOFs/TiO_2_ unchanged but reducing the concentration level for levofloxacin hydrochloride solution also led to an increased removal rate. This phenomenon can be attributed to enhanced exposure and involvement of active sites during the adsorption process with greater amounts of added Zn/Cr-MOFs/TiO_2_.

**Figure 6:**
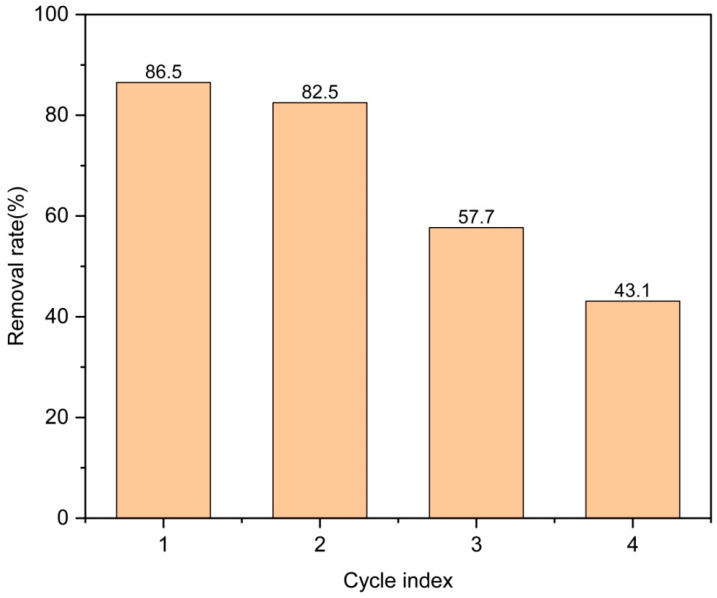
Recycling of Zn/Cr-MOFs/TiO_2_ for the removal of levofloxacin. (The errors of concentration and mass are less than ±5% and ±1%, respectively.).

The removal repeatability of Zn/Cr-MOFs/TiO_2_ for levofloxacin was investigated by adding 30 mg Zn/Cr-MOFs/TiO_2_ into a 100 ppm levofloxacin solution. After four cycles, the removal rate remained at 43.1% (as depicted in Figure 6). This demonstrates the potential reusability of Zn/Cr-MOFs/TiO_2_ for levofloxacin removal.

### 2.3. Adsorption Equilibrium

**Figure 7:**
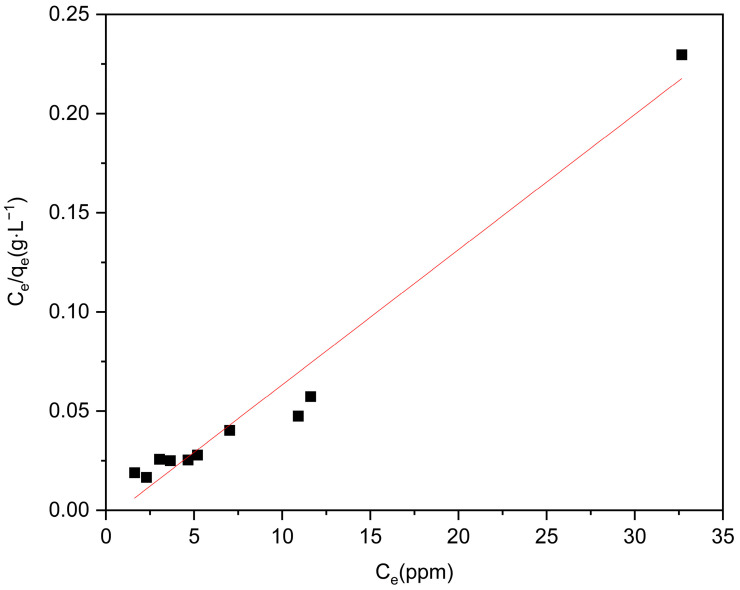
Langmuir isotherm of levofloxacin hydrochloride onto Zn/Cr-MOFs/TiO_2_.

**Figure 8:**
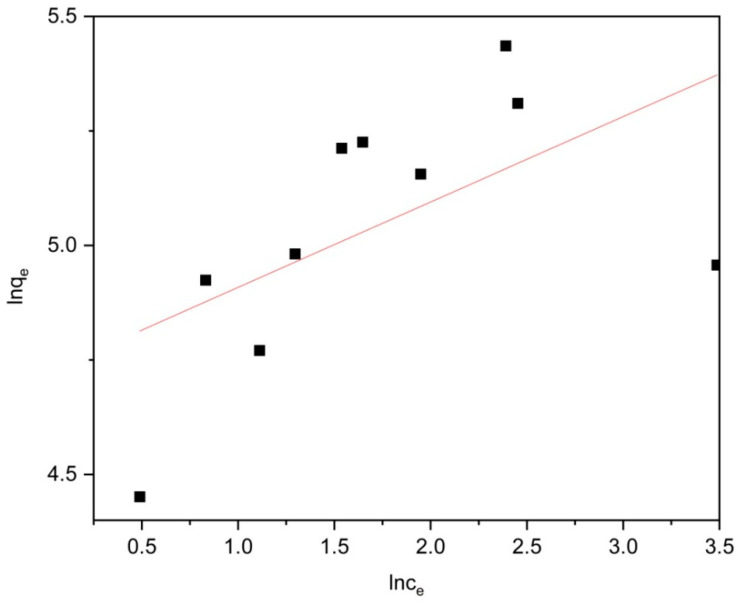
Freundlich isotherm of levofloxacin hydrochloride onto Zn/Cr-MOFs/TiO_2_.

To further validate the experimental findings, we employed Langmuir and Freundlich isotherm models to analyze the collected data. As shown in Figure 7 and Figure 8, and Table 1, the determination coefficients (R^2^) for Langmuir and Freundlich were determined to be 0.96188 and 0.2426, respectively. It can be observed that the adsorption of levofloxacin hydrochloride by Zn/Cr-MOFs/TiO_2_ follows a monolayer adsorption pattern based on the higher consistency exhibited by the Langmuir isotherm model.

### 2.4. Kinetic Modeling

In this study, we employed pseudo-first and pseudo-second-order kinetic models to accurately depict the adsorption process of levofloxacin hydrochloride by Zn/Cr-MOFs/TiO_2_. The two-stage kinetic model was initially proposed in the 1960s by Irish physicists Conan and Macquarrie. They categorized the participating particles of a multibody system into two distinct states, referred to as “active” and “dormant”. As per their definition, active particles exhibit rapid interconversion between different chemical species, while dormant particles do not possess this capability. Over time, active particles can transition into a dormant state, whereas dormant particles can also undergo activation. These models effectively illustrate the temporal changes in concentration of levofloxacin hydrochloride during adsorption. By comparing experimental data with model predictions, we can assess the level of agreement between theory and practice, shedding light on the underlying mechanism behind Zn/Cr-MOFs/TiO_2_’s adsorption of levofloxacin hydrochloride. The dynamic formulas utilized are as follows:

(1) $$ln\;C_{t}/C_{0}={-k}_{1}t$$

(2) $$\frac{t}{q_{t}}=\frac{t}{q_{e}}+\frac{1}{k_{2}q_{e}^{2}}$$

In these equations, C_t_ represents the concentration of levofloxacin hydrochloride at time t, C_0_ denotes the initial concentration of levofloxacin hydrochloride, k_1_ and k_2_ represent the rate constants (L·min^−1^ and g·(mg·min)^−1^) for the kinetic reaction, and t signifies the reaction time (min). The adsorbent’s concentration (mg/g) at time t and equilibrium is denoted as qt and qe, respectively.

**Figure 9:**
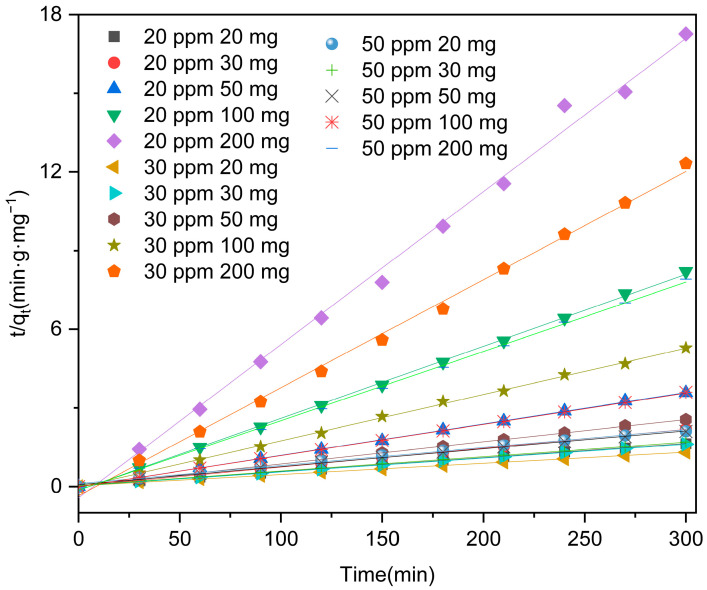
Kinetic model for the adsorption levofloxacin hydrochloride over the Zn/Cr-MOFs/TiO_2_ (linear).

**Figure 10:**
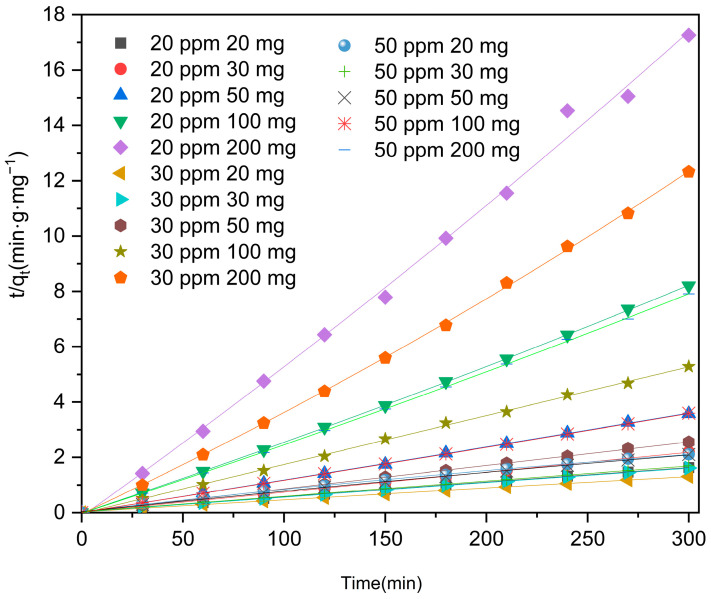
Kinetic model for the adsorption levofloxacin hydrochloride over the Zn/Cr-MOFs/TiO_2_ (non-linear).

Based on Figure 9 and Figure 10, Table 2 findings, it can be observed that the pseudo-second kinetic model exhibits a higher R^2^ value compared to that of the pseudo-first kinetic model, indicating a stronger agreement between simulation and experimental results. This suggests that Zn/Cr-MOFs/TiO_2_ primarily follows second-order kinetics in terms of levofloxacin hydrochloride adsorption.

### 2.5. Role of pH and Temperature

**Figure 11:**
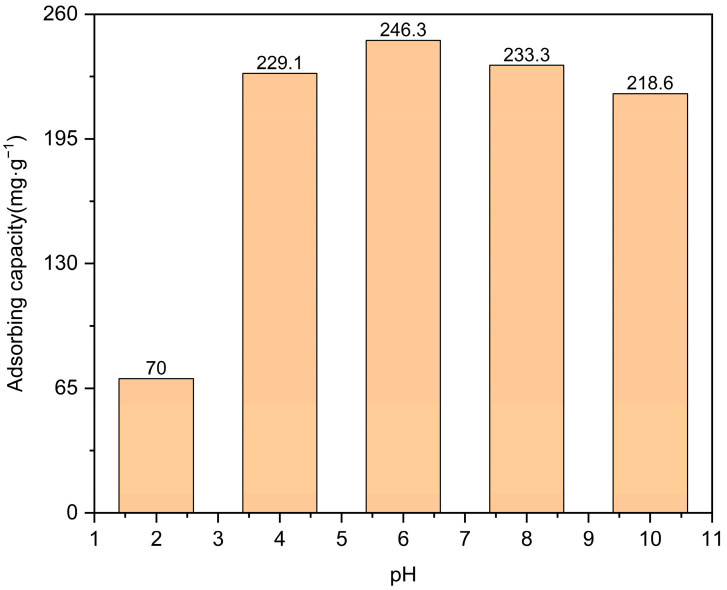
The impact of pH on the adsorption capacity of levofloxacin hydrochloride. (The error of adsorption capacity is less than ±1%).

To investigate the influence of pH on the adsorption behavior of levofloxacin hydrochloride, a 20 mg Zn/Cr-MOFs/TiO_2_ material was introduced into a levofloxacin hydrochloride solution with varying pH levels (pH = 2, 4, 6, 8, 10). As illustrated in Figure 11, an increase in pH value leads to an augmentation in the adsorption capacity of levofloxacin hydrochloride. The highest adsorption capacity is achieved at pH 6. This phenomenon can be primarily attributed to electrostatic interactions between charges present in both Zn/Cr-MOFs/TiO_2_ and levofloxacin hydrochloride molecules, resulting in optimal substance adsorption per unit mass or volume. The zeta potential characterization indicated that the charge in Zn/Cr-MOFs/TiO_2_ was 0.19 mV in water, which could be adsorbed electrostatically [34].

To investigate the impact of temperature on the adsorption behavior of levofloxacin hydrochloride by Zn/Cr-MOFs/TiO_2_, a solution containing 30 ppm levofloxacin hydrochloride was treated with 20 mg of Zn/Cr-MOFs/TiO_2_ material. The influence of different temperatures (T = 30 °C, 50 °C, 80 °C) on the adsorption efficiency of levofloxacin hydrochloride by Zn/Cr-MOFs/TiO_2_ was examined. The experimental procedure is described as follows:

(3) $$lnK_{0}=\frac{∆S^{0}}{R}-\frac{∆H^{0}}{RT}$$

(4) $$∆G^{0}=-RTlnK_{0}$$

(5) $$K_{0}=\frac{q_{e}}{c_{e}}$$

In this study, we employed the ideal gas constant (R = 8.314 J mol^−1^ K^−1^) and Langmuir adsorption constant (K_0_ in L/mol). To determine the values of ΔH^0^ and ΔS^0^, a van’t Hoff plot was utilized by plotting lnK_0_ against 1/T and conducting linear regression analysis. Specifically, ΔH^0^ was calculated as the negative slope multiplied by R, while ΔS^0^ was obtained by multiplying the intercept with R.

**Figure 12:**
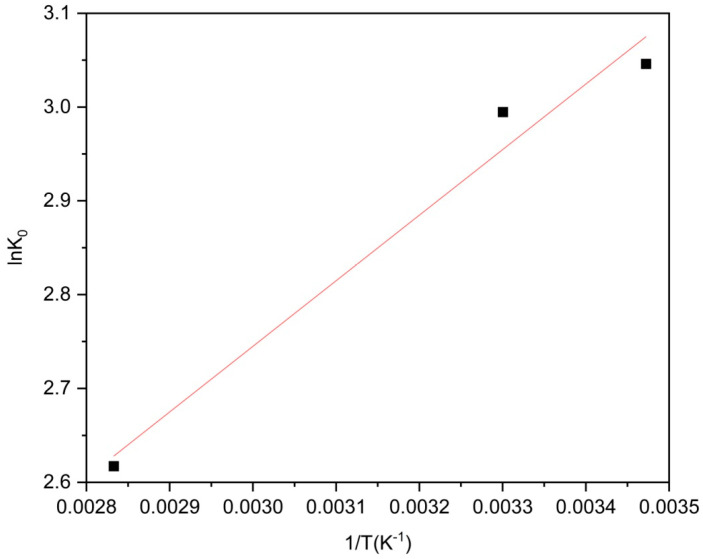
Van’t Hoff plots to obtain the ΔH and ΔS of levofloxacin hydrochloride adsorption over Zn/Cr-MOFs/TiO_2_.

The experimental results depicted in Figure 12 and Table 3 unequivocally demonstrate negative values for both ΔG^0^ and ΔH^0^, signifying an exothermic as well as spontaneous adsorption phenomenon of levofloxacin hydrochloride on Zn/Cr-MOFs/TiO_2_. The underlying mechanism encompasses a blend of physical and chemical processes wherein enthalpy fluctuations ranging from 84 to 420 kJ/mol are mainly attributed to chemisorption [35,36]. Physical adsorption becomes apparent when there is an enthalpic change below 84 kJ/mol [37].Henceforth, it can be deduced that the principal mode governing levofloxacin hydrochloride’s interaction with Zn/Cr-MOFs/TiO_2_ entails physical associations. As such, the intricate interplay between entropy and enthalpy significantly influences the overall adsorptive behavior exhibited by levofloxacin hydrochloride towards Zn/Cr-MOFs/TiO_2_ [38,39,40]. Considering these factors collectively, it can be concluded that the driving force behind levofloxacin hydrochloride adsorption onto Zn/Cr-MOFs/TiO_2_ predominantly arises from physical interactions.

**Figure 13:**
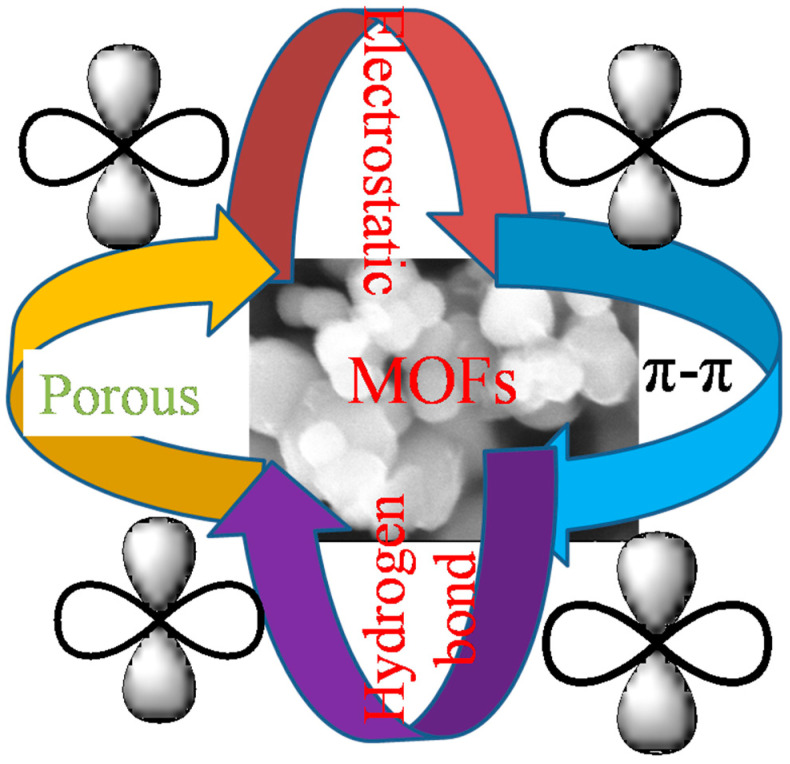
Adsorption mechanism of Zn/Cr-MOFs/TiO_2_ on levofloxacin.

In this study, we conducted a comparative analysis of the adsorption capacity of Zn/Cr-MOFs/TiO_2_ for levofloxacin hydrochloride and presented the findings in a tabular format (as shown in Table 4). The UiO-66/CoSO_4_ composite, synthesized by Zhu et al. [41] exhibits a simulated adsorption capacity of 108.4 mg/g according to the adsorption isotherm analysis. The 3% Co-MCM-41 material, prepared by Jin et al. [42,43] demonstrates an adsorption capacity of 108.1 mg/g at pH 8.5, with an adsorbent dosage of 1 g/L and an initial levofloxacin hydrochloride concentration of 119.8 mg/L. Yu et al. [44]. successfully utilized zirconium ion-modified corn bracts (CBs) as agricultural waste for efficient adsorption, achieving a maximum adsorption capacity of 73 mg/g at pH 11. We integrated a kinetic model, an isothermal equation, the mass of Zn/Cr-MOFs/TiO_2_, and the influence of levofloxacin hydrochloride concentration. As shown Figure 13, the adsorption process between Zn/Cr-MOFs/TiO_2_ and levofloxacin hydrochloride can be attributed to several factors: Firstly, both Zn/Cr-MOFs/TiO_2_ and levofloxacin hydrochloride contain benzene rings that facilitate their interaction through π-π bonding. Secondly, by observing the adsorption and desorption behavior of Zn/Cr-MOFs/TiO_2_ under an Ar atmosphere, it becomes evident that its large specific surface area and pore size enable effective adsorption of levofloxacin hydrochloride molecules within its pores. Thirdly, pH experiments reveal the presence of free groups on the surface of Zn/Cr-MOFs/TiO_2_, which can interact with levofloxacin hydrochloride via net charge attraction. Lastly, hydrogen bonding may also contribute to the mechanism underlying the adsorption between Zn/Cr-MOFs/TiO_2_ and levofloxacin hydrochloride. These factors collectively lead us to conclude that Zn/Cr-MOFs/TiO_2_ exhibits a favorable capability for effectively adsorbing levofloxacin hydrochloride [45,46,47].

## 3. Experimental

### 3.1. Experimental Reagents and Instruments

The compound 2,5-dihydroxyterephthalic acid was obtained from Shanghai Haohong Bio-Pharmaceutical Technology Co., Ltd. (Shanghai, China). The procurement of titanium dioxide and zinc acetate dihydrate was conducted through Shanghai Aladdin Biochemical Technology Co., Ltd. (Shanghai, China). Furthermore, the acquisition of chromium acetate was facilitated by Shanghai Yien Chemical Technology Co., Ltd. (Shanghai, China).

To investigate the structure of Zn/Cr-MOFs/TiO_2_ composites, we employed a range of advanced instruments for material characterization. The crystal structure of Zn/Cr-MOFs/TiO_2_ composites was analyzed using the TD-3300 XRD diffractometer, Nippon Electronics’ JSM-6700F mode field emission scanning electron microscope, Shimadzu Company’s IRAffinity-1 infrared spectrometer, and the DTG-60 differential thermogravimetric synchronization analyzer, respectively (Shimadzu, Tokyon, Japan). Additionally, Shanghai Meisei Instrument Co., Ltd.’s’ UV-8000S double beam UV-visible spectrophotometer enabled us to measure Levofloxacin hydrochloride concentration at different time points (Shanghai, China).

### 3.2. Preparation of Zn/Cr-MOFs/TiO_2_ Composites

In a 75 mL reactor, accurately weigh 2,5-dihydroxyterephthalic acid (0.0015 mol, 0.3000 g), chromium acetate (0.3469 g, 0.0015 mol), and zinc acetate dihydrate (0.0015 mol, 0.3324 g). Add 45 mL of DMF as the organic solvent for dissolution. Subsequently, introduce titanium dioxide (0.01500 g) into the same reactor and stir for 2 min before placing it in a constant-temperature drying oven at 150 °C for a duration of 12 h to initiate the reaction process. After completion of the reaction, allow the reactor to cool down to room temperature, followed by natural filtration to separate the reaction products from any impurities present in the solution. Perform one wash with filtrate and then transfer the product obtained onto a drying oven set at a temperature of 60 °C for an additional period of time lasting up to twelve hours.

### 3.3. Study on Removal of Levofloxacin Hydrochloride by Zn/Cr-MOFs/TiO_2_ Composites

In order to investigate the adsorption capabilities of Zn/Cr-MOFs/TiO_2_ composites towards levofloxacin hydrochloride, experiments were conducted by immersing different quantities (20 mg, 30 mg, 50 mg, 100 mg, and 200 mg) of Zn/Cr-MOFs/TiO_2_ composites into a solution with varying concentrations (20 mg/L, 30 mg/L, and 50 mg/L) of levofloxacin hydrochloride. The mixtures were gently stirred under ambient lighting conditions. Samples were collected at regular intervals of every thirty minutes and analyzed for the concentration of levofloxacin hydrochloride using an ultraviolet spectrometer. The calculation formula used for analysis remained unchanged.

(6) $$q_{e}=\frac{{(C}_{0}-C_{e})V}{m}$$

(7) $$removal\;extent\left(\%\right)=\frac{\left(C_{0}-C_{t}\right)}{C_{0}}\times 100\%$$

In the provided equation, C_0_ represents the initial concentration of levofloxacin hydrochloride, C_e_ denotes the equilibrium adsorption concentration, C_t_ signifies the concentration at a specific time point t, V indicates the volume of the solution, and m refers to the mass of Zn/Cr-MOFs/TiO_2_.

## 4. Conclusions

In this study, successful synthesis of Zn/Cr-MOFs/TiO_2_ materials was achieved, and their structural properties were analyzed using techniques such as FTIR, SEM, XRD, TG, etc. These characterized materials were utilized for the efficient removal of levofloxacin hydrochloride. Experimental findings revealed that at a concentration of 30 ppm for the levofloxacin hydrochloride solution, employing 20 mg of MOFs/TiO_2_ material with a pH value of 10 resulted in a remarkable maximum adsorption capacity of 246.3 mg/g. Furthermore, when the concentration was reduced to 20 ppm and the mass increased to 50 mg for Zn/Cr-MOFs/TiO_2_ material, an impressive removal rate of 88.8% for levofloxacin hydrochloride was achieved. The theoretical analysis based on these experimental results suggests that physical adsorption is primarily responsible for the effective elimination process by Zn/Cr-MOFs/TiO_2_. Consequently, it can be inferred that Zn/Cr-MOFs/TiO_2_ holds significant potential in addressing environmental concerns associated with levofloxacin hydrochloride.

## Figures and Tables

**Table 1:** Summary of Langmuir and Freundlich isotherm constants for the removal of levofloxacin hydrochloride by Zn/Cr-MOFs/TiO_2_.

Adsorbent	Langmuir Isotherm		Freundlich Isotherm	
	K	R^2^	n	R^2^
MOFs/TiO_2_	0.00681	0.96188	5.3636	0.2426

**Table 2:** The PSO Kinetic parameters for the adsorption of levofloxacin hydrochloride over the Zn/Cr-MOFs/TiO_2_ (linear).

Concentration (mg·L^−1^, ≤±10%)	Mass (mg, <±1%)	Linear		Non-Linear	
		k_2_ (g·(mg·min)^−1^)	R^2^	k_2_ (g·(mg·min)^−1^)	R^2^
20	20	0.00535	0.99916	-	0.99919
	30	0.00721	0.99968	-	0.99964
	50	0.01198	0.99967	-	0.99971
	100	0.02744	0.99901	-	0.99993
	200	0.05838	0.99465	-	0.99522
30	20	0.00427	0.99884	-	0.99894
	30	0.00527	0.99921	-	0.9992
	50	0.00847	0.99982	-	0.9998
	100	0.01766	0.99914	-	0.99904
	200	0.04121	0.99708	-	0.99962
50	20	0.00686	0.99401	-	0.99875
	30	0.00551	0.99881	-	0.99871
	50	0.00683	0.99783	-	0.99894
	100	0.01192	0.99984	-	0.99996
	200	0.02643	0.99903	-	0.99985

**Table 3:** The thermodynamic parameters of levofloxacin hydrochloride over the Zn/Cr-MOFs/TiO_2_.

T (K)	ΔG° (kJ/mol)	ΔH° (−Slope × R) (KJ/mol)	S° (Intercept × R) (J/mol/K)
288	−4.27	−5.81	5.38

**Table 4:** Comparison of the adsorption capacity of different adsorbents for Levofloxacin hydrochloride removal.

Adsorbents	***q***_***max***_ (mg g^−1^)	References
MOFs/TiO_2_	246.3	This work
Zn/Zr-MOFs	229.6	[48]
UiO-66/CoSO_4_	108.4	[40]
MCM-41	35.5	[41]
3% Co-MCM-41	108.1	[42]
Zr-modified CBs	73.1	[43]

## Data Availability

The data that support the findings of this study are available from the corresponding author upon reasonable request.
